# Prevalence and risk factors of *Toxoplasma gondii* and *Leishmania* spp. infections in apparently healthy dogs in west Shewa zone, Oromia, Ethiopia

**DOI:** 10.1186/s12917-021-02992-w

**Published:** 2021-08-25

**Authors:** Endrias Zewdu Gebremedhin, Edilu Jorga Sarba, Getachew Kebebew Tola, Solomon Shiferaw Endalew, Lencho Megersa Marami, Asaminew Tesfaye Melkamsew, Vincenzo Di Marco Lo Presti, Maria Vitale

**Affiliations:** 1 Department of Veterinary Sciences, Ambo University, School of Veterinary Medicine, P. O. Box 19, Ambo, Ethiopia; 2 Department of Veterinary Laboratory Technology, Ambo University, School of Veterinary Medicine, P. O. Box 19, Ambo, Ethiopia; 3 National Animal Health Diagnostic and Investigation Center, P. O. Box 04, Sebeta, Ethiopia; 4 Istituto Zooprofilattico sperimentale della Sicilia “A. Mirri”, Area Territoriale, Barcellona Pozzo di Gotto, Italy; 5 Laboratory of Genetics of Microorganisms, Istituto Zooprofilattico Sperimentale of Sicily, Via Gino Marinuzzi 3, 90129 Palermo, Italy

**Keywords:** Dogs, Ethiopia, *Leishmania*, Risk factors, Seroprevalence, *T. Gondii*

## Abstract

**Background:**

In urban settings, the presence of a high density of the human population and contact with domestic and/or stray animals such as dogs and cats can be risk factors for the transmission of zoonotic protozoa parasites. *Toxoplasma gondii* (*T. gondii*) and *Leishmania* spp. are zoonotic protozoon parasites with significant health burdens worldwide.

**Methods:**

A cross-sectional study was used to investigate the antibody prevalence and risk factors of *T. gondii* and *Leishmania* spp. infections in 385 randomly selected dogs of Ambo, Bako, and Gojo towns of West Shewa Zone, Oromia regional state, Ethiopia. A questionnaire survey was administered to households to collect data on potential risk factors. Dog sera samples were assayed for *T. gondii* IgG antibodies using the direct agglutination test while *Leishmania* spp. specific antibodies tested using an indirect enzyme-linked immunosorbent assay (ELISA). Logistic regression was used for data analysis.

**Results:**

Overall, *T. gondii* and *Leishmania* spp. infection seroprevalence was found to be 82.86% (95% confidence interval (CI): 78.71–86.49%) and 92.47% (95% CI: 89.36–94.90%), respectively. Seropositivity for both *T. gondii* and *Leishmania* spp. was found in 82.58% of the dogs. None of the investigated factors were associated with *Leishmania* spp. seropositivity (*p* > 0.05). The seroprevalence of *T. gondii* was significantly different among the study towns (*p* = 0.003). The risk of *T. gondii* infection was 2.71 times higher in adult dogs than juvenile dogs (*p* = 0.043). Dogs kept simultaneously with other domestic animals had increased odds of *T. gondii* seropositivity compared to those with no other domestic animals (Adjusted Odds ratio: 1.96, *p* = 0.021). However, altitude, sex, breed, housing, feeding, educational level of head of the household, and dog’s living area were not significantly associated with *T. gondii* seropositivity (*p* > 0.05).

**Conclusions:**

The high seropositivity and the simultaneous presence of antibodies of *T. gondii* and *Leishmania* spp. in dogs suggest the widespread nature of these parasites in the environment and the high potential of transmission to other animals and humans. Further epidemiological studies, isolation and molecular characterization of the parasites, and educational campaigns are suggested.

## Introduction

Toxoplasmosis and leishmaniasis are important zoonotic diseases both caused by unicellular parasites. Toxoplasmosis is spread worldwide since *T. gondii* can infect almost all warm-blooded animals and humans and can be transmitted through many different routes [1]. Leishmaniasis is a vector-borne disease of great medical and veterinary importance in different geographic areas caused by different *Leishmania* spp. transmitted by sand fly vectors (*Phlebotomus* spp.). There are two major clinical forms of the disease in both humans and dogs, cutaneous (CL) and visceral leishmaniasis (VL, also known as Kala-azar) [2]. Leishmaniases are neglected tropical and subtropical diseases endemic to 98 countries worldwide [3] including Ethiopia [4]. Visceral leishmaniasis (VL) affects about 12 million people worldwide with almost 0.5 million new cases of VL and 350 million people are at risk of infection each year [5]. Ethiopia, India, Bangladesh, Sudan, South Sudan, and Brazil are countries with a high prevalence of visceral leishmaniasis (90% of cases) [3]. Canine Leishmaniasis (Can L) is a very serious disease when remains untreated and can be a focus of transmission to other dogs or humans particularly in endemic areas where the sandflies are present. Canine leishmaniasis is an endemic disease in more than 70 countries and is a common disease in the Mediterranean region. However, many cases have been reported in non-endemic areas, like the United Kingdom, Germany, and Poland, where this disease is considered exotic [6] The cases in North-European countries are probably related to the owners’ traveling with their dogs exposed to competent insect vectors through endemic regions. In addition, non-vectorial transmission between dogs including infection through transfused blood products from infected donors, transplacental and venereal transmission have been reported [7].

Upon parasite transmission, some dogs can control the infection for many years, without the appearance of clinical signs while other dogs may display an acute evolution and severe disease, or progressive course that leads to death if proper management and therapy are not adopted. The management of CanL is being performed recently using prophylactic measures in healthy dogs such as using insecticides impregnated collars [8].

In Ethiopia, there are several foci of *Leishmania* spp. infections in humans with frequent outbreaks leading to over 7000 and 50,000 new cases of visceral (VL) and cutaneous leishmaniasis (CL) per year, respectively [9]. This has contributed to their identification as a major public health concern. Leishmaniasis, however, remains one of the most overlooked tropical diseases [10]. Many infected animals are asymptomatic in endemic areas, and their role in infection transmission is mainly unknown [11]. The prevalence of infection in dogs is high and they represent urban domestic reservoirs for *Leishmania* spp. playing an essential role in disease epidemiology [12]. In Ethiopia, dogs and hyraxes are the main reservoir hosts for visceral and cutaneous leishmaniasis, respectively [9]. However, poor knowledge of canine leishmaniasis in the Ethiopian dog populations is available. As part of a study investigating the human VL outbreak in Libo Kemkem, Ethiopia, Alvar et al. [13] reported *Leishmania* DNA in the venous blood of two of the 40 asymptomatic dogs sampled. In northwest Ethiopia, where foci of human VL are common, Kalayou et al. [14] reported an overall seroprevalence of *L. donovani* infection of 27.7 and 14.8% in dogs, using direct agglutination test and Kala-azar detect rapid test, respectively. However, the dog (*C. familiaris*) population in Ethiopia is unknown and data on dog-related zoonotic diseases is scarce. Dog holding in big cities in Ethiopia has increased significantly in recent years along with increased urbanization. Dogs are mainly kept to protect owners and household properties. However, the attitude of keeping dogs as companion animals is also growing with the presently rising trend of urbanization and customizing western culture. In Addis Ababa, some people generate income by breeding and selling exotic dog breeds [15].

*T. gondii* can infect almost all warm-blooded animals and humans and can be transmitted through many different routes *T. gondii* is one of the most common parasites on earth, infecting as much as one-third of the world’s human population [16]. The health burden of toxoplasmosis has been ranked among the highest of all parasitic diseases [17]. Humans are infected by *T. gondii* when they are consuming undercooked intermediate host meat harboring cysts, drinking oocyst-contaminated water by the final host (felids), and through congenital transmission. Only a small percentage of infected people exhibit clinical symptoms of the disease. *T. gondii* infection in pregnant women, on the other hand, can cause severe and disabling disease in the developing fetus [16, 18]. Subclinical and clinical infections with *T. gondii* including fatal cases have been described in dogs [16].

Stray dogs and owned dogs with outdoor access play an important role in the epidemiology of *T. gondii* infection. This is due to the practice of feeding dogs on food types from various sources like garbage and food contaminated with soil. Like cats, dogs may also serve as a possible source of *T. gondii* infection in humans due to close contact [19]. Human *T. gondii* oocysts exposure through dogs can occur in connection with the mechanical transport of oocysts from the feces of cats by rolling in foul-smelling substances [20] thus serving as the parasite’s environmental sentinel [18]. Dogs can become infected by the ingestion of *T. gondii* oocysts from cat feces or by the feeding habit of uncooked mutton (carnivorism). Antibodies to *T. gondii* were found worldwide in canine sera and viable *T. gondii* were also segregated from dogs’ muscles and brain tissues [16, 18].

In Ethiopia, a meta-analytical study of IgG seroprevalence for *T gondii* found a high pooled prevalence in animals (87.72% in cats, 34.59% in small ruminants) and humans (74.73%) with a high risk of sheep and goat reproductive problems and multiple human diseases [21]. However, no single published information is available about infection in dogs so far.

Toxoplasmosis in dogs is typically asymptomatic, and the clinical process in the respiratory and hepatic systems is often most noticeable when it occurs. Clinical cases of toxoplasmosis in cats are much more common than in dogs. A high proportion of clinical infections with *T. gondii* are caused by immunosuppressive chemotherapy [22]. However, neurological symptoms have also been identified [23, 24]. The clinical type may be due to the reactivation of latent infection associated with the immunosuppression caused by the canine distemper virus [25].

Good knowledge of the prevalence of *T. gondii* and *Leishmania* spp. in household dogs may aid in designing and implementing appropriate disease management strategies and could therefore benefit both animal and human health. Therefore, the present study was aimed to estimate the seroprevalence and associated risk factors of *T. gondii* and *Leishmania* spp. infections in dogs in Ambo, Bako, and Gojo towns of West Shewa Zone, Oromia, Ethiopia.

## Results

### Seroprevalence

The overall seroprevalence of *T. gondii* infection in dogs was found to be 82.86% (319/385, 95% confidence interval [CI]: 78.71–86.49%), and it was significantly different among the studied towns (*Χ*^2^ = 13.72, *p* = 0.003). Of 385 dogs’ sera tested for anti-*Leishmania* spp. antibodies, 356 (92.47, 95% CI: 89.36–94.90%) were seropositive with no statistically significant difference among the analyzed towns (*Χ*^2^ = 0.92, *p* = 0.632). There was no statistically significant association between *Leishmania* spp. seropositivity and the independent variables evaluated in the study (*p* > 0.05) (Table 1).

**Table 1:** The overall seroprevalence of *T. gondii* and *Leishmania* spp. infection in dogs of the study towns

Town	No. tested	T. gondii*		*Leishmania* spp.	
		No. positive	% prevalence (95% CI)	No. positive	% prevalence (95% CI)
Ambo	169	127	75.15 (67.93–81.46)	157	92.90 (87.93–96.28)
Gojo	68	59	86.76 (76.36–93.77)	61	89.71 (79.93–95.76)
Bako	148	133	89.86 (83.83–94.22)	138	93.24 (87.93–96.71)
Overall	385	319	82.86 (78.71–86.49)	356	92.47 (89.36–94.90)

*Pearson Chi^2^ (3) = 13.72, *p* = 0.003, CI = Confidence interval

Age-wise, the highest seroprevalence of *T. gondii* infection was found in adult dogs (84.35%). The presence of cats and other domestic animals in the household was significantly associated with *T. gondii* seroprevalence (Table 2).

**Table 2:** Results of logistic regression analysis of *T. gondii* prevalence and potential risk factors in selected districts of West Shewa zone, Ethiopia

Variable	Categories	No. tested	No. pos. (% prevalence)	Univariable		Multivariable	
				OR (95% CI)	P	OR (95% CI)	P
Town	Ambo	169	127 (75.15)	1.0		1.0	
	Gojo	68	59 (86.76)	2.17 (0.99–4.75)	0.053		
	Bako	148	133 (89.86)	2.93 (1.55–5.55)	0.001*		
Altitude	Highland (≥2100 masl)	237	186 (78.48)	1.0		1.0	
	Midland (1600–2100 masl)	148	133 (89.86)	2.43 (1.31–4.51)	0.005*	2.36 (1.23–4.50)	0.009*
Age	Juvenile	27	19 (70.37)	1.0		1.0	
	Adolescent	77	63 (81.82)	1.89 (0.69–5.20)	0.214	2.42 (0.83–7.03)	0.105
	Geriatrics	51	43 (84.31)	2.26 (0.74–6.93)	0.153	2.77 (0.85–8.97)	0.090
	Adult	230	194 (84.35)	2.27 (0.92–5.59)	0.074	2.85 (1.09–7.43)	0.032*
Sex	Male	293	239 (81.57)	1.0		1.0	
	Female	92	80 (86.96)	1.51 (0.77–2.96)	0.234	1.62 (0.79–3.32)	0.186
Breed	Exotic	15	11 (73.33)	1.0		1.0	
	Cross	74	61 (82.43)	1.71 (0.47–6.21)	0.417		
	Indigenous	296	247 (83.45)	1.83 (0.56–5.99)	0.316		
Feeding	Cooked	103	83 (80.58)	1.0	–		
	Raw animal products	282	236 (83.69)	1.24 (0.69–2.21)	0.475		
Housing	Indoor	119	93 (78.15)	1.0		1.0	
	Outdoor	106	87 (82.08)	1.28 (0.66–2.48)	0.463	1.44 (0.71–2.90)	0.309
	Mixed	160	139 (86.88)	1.85 (0.98–3.48)	0.056	1.55 (0.79–3.03)	0.203
PODAHH	No	181	142 (78.45)	1.0		1.0	
	Yes	204	177 (86.76)	1.80 (1.05–3.08)	0.032*	1.94 (1.10–3.42)	0.022*
Education of HHH	Secondary	125	100 (80.00)	1.0		1.0	
	Illiterate	47	38 (80.85)	1.06 (0.45–2.47)	0.901	1.02 (0.42–2.48)	0.957
	Tertiary	115	96 (83.48)	1.26 (0.65–2.44)	0.487	1.33 (0.65–2.70)	0.435
	Primary	98	85 (86.73)	1.63 (0.79–3.39)	0.187	1.50 (0.70–3.19)	0.298
Living area/residence	Urban	341	280 (82.11)	1.0			
	Peri-urban	44	39 (88.64)	1.70 (0.64–4.49)	0.285		
Presence of cats in the household	No	214	170 (79.44)	1.0		1.0	
	Yes	171	149 (87.13)	1.75 (1.00–3.06)	0.048*	1.65 (0.92–2.95)	0.094
Family size of a dog-owning household	≤ 4	114	93 (81.58)	1.0			
	≥ 5	271	226 (83.39)	1.13 (0.64–2.01)	0.666		
HHH	Protestant	248	199 (80.24)	1.0			
	Orthodox	118	102 (86.44)	1.57 (0.85–2.90)	0.149		
	*Waqefeta*	8	7 (87.50)	1.72 (0.21–14.34)	0.614		
	Muslim	11	11 (100.00)	–	–		
Marital status of dog-owning HHH	Divorce	25	20 (80.00)	1.0			
	Married	343	283 (82.51)	1.18 (0.43–3.27)	0.751		
	Single	17	16 (94.12)	4.00 (0.42–37.78)	0.226		

* Statistically significant

PODAHH = presence of other domestic animals in the household, HHH = head of the household, RHHH = religion of the head of the household

Full model = HLX2 = 7.70, P-Value = 0.4632, Se = 99.37, Sp = 1.52, PPV = 82.98, NPV, 33.33, ROC = 0.6993

Best fitting model = HLX2 = 5.89, P-Value = 0.6594, Se = 99.37, Sp = 3.03, PPV = 83.20, NPV, 50.0, ROC = 0.6741

**Fig. 1:**
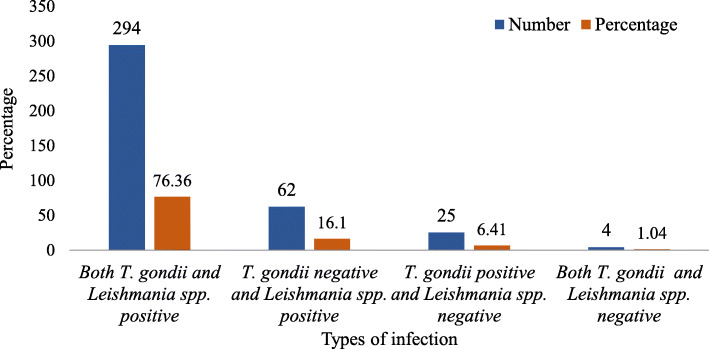
The rate of co-infection of *T. gondii* and *Leishmania* spp. in dogs of the study towns

The study revealed that 82.58% (*n* = 294) of the studied dogs were seropositive for both *T. gondii* and *Leishmania* spp. (Fig. 1). The Goodman and Kruskal’s gamma statistics for correlation between the two binary outcome variables (*Toxoplasma gondii* and *Leishmania* spp. seropositivity) was weak and negative, which is not statistically significant (Goodman and Kruskal’s gamma statistics value = − 0.137 *p* = 0.591).

### Risk factors

#### *Toxoplasma gondii* infection

As indicated in Table 2 below, univariable logistic regression analysis showed that the likelihood of *T. gondii* seropositivity was 2.93 times higher in dogs of Bako town as compared to Ambo (*p* = 0.001). Similarly, the risk of *T. gondii* seropositivity in dogs was 1.80 times higher in households where other domestic animals are found (*p* = 0.032). The *T. gondii* seropositivity of dogs is significantly associated with the presence of cats in dog-owning households (OR = 1.75, 95% CI: 1.00–3.06, *p* = 0.048).

In the multivariable logistic regression analysis, the risk of *T. gondii* infection in adult dogs was 2.71 times higher than in juvenile dogs (*p* = 0.043). The likelihood of getting seropositive dogs was 1.96 times high in households where other domestic animals are present than when they were absent (*p* = 0.021). Thus, the age of dogs and the presence of other domestic animals in the household were independent predictors of *T. gondii* seropositivity. On the other hand, altitude, sex, housing, and presence of cats in the household showed no significant association with *T. gondii* seropositivity in the final model (*p >* 0.05) (Table 2).

#### *Leishmania* spp. infection

All independent variables investigated were non-collinear with each other except district vs altitude (r = − 0.87). Based on the univariable logistic regression analysis, dogs’ lifestyle, community type, and presence of cats in the household were variables that had *p* < 0.25 and hence they were selected for the multivariable model. As a result, none of the risk factors investigated were independent predictors of *Leishmania* spp. infection (p > 0.05) (Table 3).

**Table 3:** Results of logistic regression analysis of seroprevalence of *Leishmania* spp. infection and potential risk factors in selected districts of West Shewa zone, Ethiopia

Variable	Categories	No. tested	No. pos. (% prevalence)	Univariable		Multivariable	
				OR (95% CI)	P	OR (95% CI)	P
Town/location	Gojo	68	61 (89.74)	1.0			
	Ambo	169	157 (92.90)	1.50 (0.56–3.99)	0.415		
	Bako	148	138 (93.24)	1.58 (0.58–4.36)	0.373		
Altitude	Highland	237	218 (92.0)	1.0			
	Midland	148	138 (93.2)	1.20 (0.54–2.66)	0.649		
Age	Adolescent	77	70 (90.91)	1.0			
	Geriatrics	51	47 (92.16)	1.18 (0.33–4.24)	0.805		
	Adult	230	213 (92.61)	1.25 (0.50–3.15)	0.631		
	Juvenile	27	26 (96.30)	2.6 (0.30–22.17)	0.382		
Sex	Female	92	85 (92.39)	1.0			
	Male	293	271 (92.49)	1.01 (0.42–2.46)	0.975		
Breed	Cross	74	68 (91.89)	1.0			
	Indigenous	296	274 (92.57)	1.10 (0.43–2.82)	0.844		
	Exotic	15	14 (93.33)	1.24 (0.14–11.08)	0.850		
Housing	Outdoor	106	95 (89.62)	1.0		1.0	
	Indoor	119	110 (92.44)	1.42 (0.56–3.56)	0.461	1.19 (0.46–3.07)	0.719
	Mixed	160	151 (94.38)	1.94 (0.78–4.86)	0.156	1.74 (0.69–4.41)	0.243
PODAHH	Yes	204	187 (91.67)	1.0		1.0	
	No	181	169 (93.37)	1.28 (0.59–2.76)	0.528		
Education of HHH	Secondary	125	113 (90.40)	1.0		1.0	
	Primary	98	90 (91.84)	1.19 (0.47–3.05)	0.710		
	Tertiary	115	108 (93.91)	1.64 (0.62–4.32)	0.318		
	Illiterate	47	45 (95.74)	2.39 (0.51–11.10)	0.266		
Living area/residence	Peri-urban	44	38 (96.36)	1.0		1.0	
	Urban	341	318 (93.26)	2.18 (0.84–5.70)	0.111	2.05 (0.77–5.47)	0.152
Presence of cats	No	214	194 (90.65)	1.0		1.0	
	Yes	171	162 (94.74)	1.86 (0.82–4.19)	0.137	1.72 (0.75–3.93)	0.197

## Discussion

In this study, the seroprevalence and risk factors for *T. gondii* and *Leishmania* spp. infections were carried out on 385 dogs to understand the epidemiology and control measures for these infections in dogs and public health interventions. The current finding revealed that the seroprevalence in apparently healthy dogs for these two important zoonotic protozoon parasites was very high.

The *T. gondii* seroprevalence (82.86%) in dogs corroborates well with the previous meta-analysis prevalence reports from Ethiopia in cats (87.72%) but is higher than the reports in small ruminants (34.59%) [21]. The high seroprevalence of *T. gondii* infection in this study indicates the widespread contamination of the urban environment with the parasite. Previous studies in seropositive sheep and goats [26], backyard chickens [27], and pigs [28] in central Ethiopia demonstrated the isolation of viable tissue cysts indicating that these animals might serve as a source of infection for dogs. In urban environments, dogs are likely to acquire *T. gondii* infection from infected prey such as birds, rodents, or tissue cysts from human leftover food available in the garbage [16, 29]. Moreover, the warm moist temperature and the high percentage of relative humidity in the studied towns might be favorable for the survival of the *T. gondii* oocysts [16].

Univariable logistic regression analysis showed a significant difference in the seroprevalence of *T. gondii* infection among the towns, i.e., it was high in Bako (*p* = 0.001) compared to Ambo town. This might indicate that climate considerably influences the risk of *T. gondii* exposure. The warm and moist environment coupled with the more abundance of cats [30] and the source of infection for dogs (cats, the meat of infected domestic or wild animals containing tissue cysts) in Bako town might explain the higher seroprevalence. It has been well documented that seroprevalence varies according to the density of cats and intermediate hosts [19], geographical location, and even within the same region from place to place. If cat-owning households there will be an ample chance to contaminate animals’ farmlands, feed, and water [16]. The results showed an increase in seroprevalence from juvenile (70.37%) to geriatrics (84.31%) stage in agreement with the previous report [31]; the odds of acquiring *T. gondii* infection in adult dogs is nearly 2.71 times higher as compared to juvenile dogs (*p* = 0.043). As the age of dogs increases the likelihood of acquiring *T. gondii* infection from the environment increases i.e., postnatal/horizontal infection is the main route of infection [16, 32–36]. Moreover, the lifelong persistence of IgG antibodies once infected might also add to the high prevalence in older dogs [16].

Very high seroprevalence of *Leishmania* spp. infection was observed in the present study (92.47%) in contrast to Rohousova et al. [37] which reported relatively lower seropositivity of 55.9% (19/34) and PCR positivity of 5.9% (2/34) in dogs of Northwestern Ethiopia (Oromia). However, in that Ethiopian region, no data are available on the competent vector populations present, so we cannot exclude that dogs might be the preferential hosts for the sand fly species present in this area. A complex relationship between hosts, parasites, and sand fly vectors, makes the transmission of *Leishmania* spp. quite intricate as also suggested by the so-called paradox of Cyprus where a high seroprevalence for *L. infantum* in the dog population does not correspond to leishmaniasis cases and seroprevalence in humans; two transmission cycles seem to run in parallel in Cyprus: in dogs with *L. infantum* and humans with *L. donovani* [38]. The expansion of agricultural activities increased urbanization, the abundance of reservoir hosts (e.g., hyraxes) and the biological vectors (sandflies) adaptation of the parasites and vectors might also contribute to the high seroprevalence [9, 14, 39]. Moreover, the weak health infrastructure and poor or absence of disease and vector control programs in dogs and humans of the current study areas, are additional contributing factors.

Although *Leishmania* infection of dogs ranging from 60 to 80% has been reported in endemic areas [40], the current seroprevalence was much higher and less related to the factors considered in this study compared to *Toxoplasma* since no statistically significant variations were detected among the three cities. This might suggest that infection transmission through a vector such as sand flies for *Leishmania* spp. might be related to environmental, structural, and human factors similar in the three cities considered in this study. Moreover, vector-borne diseases are influenced by environmental changes and socioeconomic factors such as sanitary conditions, malnutrition, population movement, or poor housing. A recent study in Nepal for human leishmaniosis in endemic districts found that houses with natural floors increased the risk of infection by eightfold, walls made from straw, leaves, and/or bamboos increased by threefold, walls with cracks, especially in the bedroom, increased by threefold and proximity to a livestock shed increased the risk by fourfold [41]. Anthropogenic factors tend to reorient the composition and behavior of sand fly vectors. To date, there are at least 50 different sand fly species transmitting leishmaniases [42].

In this study, contrary to our expectation, there was no significant difference in the seroprevalence of *T. gondii* and *Leishmania* spp. infections between indoor and outdoor kept dogs. In Ethiopia, exotic and crossbred dogs are mostly kept indoors while indigenous dogs live outdoors. However, the infection rate of both parasites was considerably high in both canine populations. For *T. gondii* infection this might be explained by the fact that both populations are fed with food waste and raw meat instead of commercial or adequately cooked food. For *Leishmania* spp. infection, the shelters for dogs are not built to avoid sandflies access and indoor conditions cannot assure the absence of the vectors. Due to the complex relationship between human, animal hosts, parasites, and sand fly vectors, the transmission of *Leishmania* spp. is intricate. Nevertheless, the absence of a statistically significant association between seroprevalence of *Leishmania* spp. and potential risk factors considered in this study should prompt further studies in the future to identify the risk factors.

There was a high percentage of concurrent infection of dogs with *T. gondii* and *Leishmania* spp. (82.58%) as well as the absence of significant difference in the seroprevalence of the two parasites across altitudes, sex, breeds, housing and living areas/residence (urban vs peri-urban). These might suggest the ubiquitous nature of the parasites and that these factors have a similar risk of infection as reported by other researchers elsewhere [35, 38, 39, 43]. Besides, the lack of association of *T. gondii* seropositivity with breed and sex of dogs might have probably be overshadowed by the high exposure to the parasite at a very young age [30, 33]. In agreement with the present study, Kalayou et al. [14] also reported the absence of a significant association between sex, housing, and place of residence and *L. donovani* seroprevalence in dogs of northwest Ethiopia.

The study identified widespread *T. gondii* and *Leishmania* spp. infections in the canine population along with the contributing risk factors for the transmission. Such information may serve in the efforts to minimize the risk of zoonosis in humans. The asymptomatically infected dogs living together or very close to humans identified in the current study might maintain *Leishmania* spp. and *T. gondii* parasites to other animals and humans. Thus, because of the high seroprevalence and the poor or non-existent veterinary medical care for dogs, high HIV/AIDS prevalence, the overall inadequate personal hygiene, and environmental sanitation in the studied towns, these zoonotic parasites might be of great public health concern since asymptomatically infected dogs might be the source of infection for humans [36].

The limitations of this cross-sectional survey include the failure to collect data on clinical manifestations of dogs to relate it with seropositivity. Nevertheless, the findings for these zoonotic parasites indicate the magnitude of infections and that dogs might be an important reservoir posing potential health risks for animals and humans.

To the best of the knowledge of the authors, this is the first report of seroprevalence of *T. gondii* infection as well as co-infection of *T. gondii* and *Leishmania* spp. from household dogs in Ethiopia.

## Conclusions

The results showed very high infection rates for both parasites and the simultaneous presence of *T. gondii* and *Leishmania* spp. in dogs suggesting the widespread nature of these parasites in the urban environments and the big potential risk of transmission to humans and other animals. The age of dogs and the presence of other domestic animals in households are predictors of *T. gondii* seropositivity. None of the investigated variables are independent predictors of *Leishmania* spp. seropositivity. Further studies to isolate, identify the genotype and virulence of the parasites, preferably from clinical cases, as well as the contribution of dogs in the transmission of the infections to humans along with hygienic measures and educational campaigns, is imperative.

## Materials and methods

### Study design and areas

A cross-sectional household survey was undertaken in Ambo, Bako, and Gojo towns of West Shewa Zone, Oromia Regional State, from January 2015 to June 2017. Ambo, Bako, and Gojo towns are the administrative centers of Ambo, Bako Tibe, and Jeldu districts, respectively. Table 4 shows the location, latitude, longitude, temperature, rainfall, elevation, and the human population of the study towns. The three towns have bimodal rainfall characterized by a short rainy season from February to May, and the large rainy season is from July to September. The dry season extends from October to January [43].

**Table 4:** Description of the study towns

Descriptions	Study towns		
	Ambo	Bako	Gojo
Distance from Addis Ababa	114 km	260 km	120 km
Latitude and longitude	8°59′N 37°51′E	9°08′N 37°03′E - 9°08′N 37°03′E	9.26′N 38.09 E
Elevation (meters above sea level)	2101	1743	2905
Average annual temperature (°C)	22	19.7	20
Average annual rainfall (mm)	900	1281	2500
Total human population [44]	74, 843	18,641	14,794

### Study population

Stray dogs were excluded from the present study. Thus, owned dogs above three months of age found in the three towns were the study population.

### Animals and samples

Domestic /owned/ dogs (*C. familiaris*) from each randomly selected “*Gotes”* (*Gote* is a subdivision of *Kebele* containing 20–30 households) were sampled from house to house. *“Kebele”* refers to the smallest administrative unit of a town. The veterinary service provided to the dogs is quite inadequate and consequently, the vast majority of the studied dogs received no rabies vaccination and /or other treatments. Dogs above three months of age were sampled to avoid transcolostral antibodies [16]. The age of dogs ranged from 3 to 168 months, with an average value of 33 months.

### Sample size and sampling technique

Since there is no previous *T. gondii* seroprevalence study in Ethiopia, 50% expected prevalence, 5% desired absolute precision, and 95% CI were used to calculate the required sample size using the formula: *N* = 1.96^2^ p_exp_ (1-p_exp_)/d^2^ [45], where n = required sample size, p = expected prevalence d = desired absolute precision. Therefore, the calculated sample size was 384. There was no accessible data on the dog population in the three towns. Thus, it was assumed that the population of dogs in the towns is evenly distributed. A multi-stage sampling procedure was employed to select households for this study. There are three, two, and one *Kebele* in Ambo, Bako, and Gojo towns respectively. From each “*Kebeles*,” four “*Gotes*” were randomly selected using the list of *Gotes* in each *Kebeles* (sampling frame) provided by local administrators. The index household in a *Gote* was randomly selected and subsequent households were surveyed door to door.

### Blood sample collection

Five milliliters of whole blood was aseptically collected from each dog’s cephalic vein using a plain vacutainer tube. The blood samples were kept at room temperature and allowed to clot in a slanted position in a cool place and serum was separated by centrifugation at 3000 RPM for 10 min, transferred into cryovials, labeled, and stored at − 20 °C until the laboratory assay was carried out.

### Questionnaire survey

A pre-tested structured questionnaire was prepared and administered to dog owners during blood sample collection. The close-ended questions asked include sex (male, female), breed (exotic, cross, indigenous), housing system (indoor, outdoor, mixed), feeding (cooked animal products, household leftover, raw animal products), presence of other domestic animals in the household (cattle, sheep, goats, horse, mule, donkey, cats, chicken), educational level of dog owner (illiterate, primary, secondary, tertiary), presence of cat/s in the household (yes, no), living area/residence (urban, peri-urban), marital status (single, married, divorced), the religion of the head of the household (Protestant, Orthodox, *Waqefeta*, Muslim), and family size of the dog-owning household (≤ 4, ≤ 5), The age of dogs was categorized as a juvenile (6 weeks to 6 months), young (6 months to 18 months), adult (18 months to 7 years), and geriatric (greater than 7 years) based on owners information [46].

### Laboratory test

Sera samples were transported to the National Animal Health and Diagnostic Center (NAHDIC) in ice packs and stored at − 20 °C until assayed. *T. gondii* IgG antibody was determined from each sample using a commercially available Direct Agglutination Test (DAT) kit (Toxo screen DA, biomerieux®, France) following the manufacturer’s instructions. Sera were assayed at a screening dilution of 1/40 and 1/4000 to avoid the false-negative results that might occur at low dilutions when using sera with high antibody titers. *T. gondii* infection was diagnosed when a serum sample gave a positive reaction indicated by a clear agglutination above half of the well at a dilution of 1: 40 or 1: 4000 or both. Sedimentation of antigen at the bottom of the well was considered as a negative result. Positive and negative controls were included in each test. All the collected serum samples were tested for the presence of antibodies against *Leishmania* spp. following the protocol of the manufacturer of the indirect ELISA kit (VetLine, NovaTec Immundiagnostica GmbH, Germany). According to the manufacturer, the sensitivity and specificity of the kit are > 98%.

### Data analysis

The findings of the questionnaire survey and laboratory data were entered into Microsoft Excel Spreadsheet. Coded data was transferred into STATA version 14.0 for Windows (Stata Corp. College Station, TX, USA). The association of the seroprevalence with putative risk factors was first statistically analyzed using Pearson’s Chi-square test. Seroprevalence figures by DAT (for *T. gondii* infection) and ELISA (for *Leishmania* spp. infection) were considered as dependent variables. Age, sex, breed, feeding, housing, town, altitude, residence place, presence of cats, presence of other domestic animals, family size, marital status, and religion were the independent/explanatory variables investigated. Univariable and multivariable logistic regressions were used to identify the predictors of *T. gondii* seropositivity. Non-collinear variables with *p*-value < 0.25 in univariable analysis were further analyzed using multivariable logistic regression to identify risk factors of seropositivity and obtain adjusted odds ratios with 95% CI. The 95% confidence level for the subgroup and overall prevalence values were calculated using the exact binomial test. Goodman and Kruskal’s gamma statistics was used to see the correlation between the binary outcomes (*T. gondii* and *Leishmania* spp. Serostatus). Differences were considered statistically significant at *p* < 0.05.

## Data Availability

The datasets used and/or analyzed during the current study are available from the corresponding author on reasonable request.
